# Distribution of genital human papillomavirus genotypes in benign clinical manifestations among men from Northern Spain

**DOI:** 10.1186/s12889-016-2713-x

**Published:** 2016-01-27

**Authors:** L. Sara Arroyo, Miren Basaras, Elixabete Arrese, Silvia Hernáez, Valentín Esteban, Ramón Cisterna

**Affiliations:** 1 Department of Laboratory Medicine, Karolinska Institutet, 14186 Stockholm, Sweden; 2 Immunology, Microbiology and Parasitology Department, School of Medicine, University of Basque Country (UPV/EHU), Barrio Sarriena s/n, 48940 Leioa, Bizkaia Spain; 3 Clinical Microbiology and Infection Control Department, Basurto University Hospital, Avda de Montevideo 18, 48013 Bilbao, Bizkaia Spain

**Keywords:** Papillomavirus infections, Condyloma acuminata, Risk factors, Epidemiology

## Abstract

**Background:**

In the literature, data on the prevalence of human papillomavirus (HPV) infection in men vary significantly and the exact distribution of specific genotypes is still unclear. As infections usually occur without symptoms, men might only attend their hospital clinic when they have a specific concern, being in most cases genital warts (condylomas), which are often caused by low-risk HPV genotypes. The aim of this study was to assess HPV genotype distribution and prevalence among men attending hospital for HPV-associated conditions and to evaluate infection-associated factors.

**Methods:**

Samples from men with clinical manifestations of HPV-related infections seen during 2007–2012 at the Clinical Microbiology and Infectious Control Department at Basurto University Hospital were genotyped using Linear Array HPV Genotyping Test kit (Roche Molecular Diagnostics, Germany). Data on probable risk factors were collected and investigated for possible association.

**Results:**

Of 184 anogenital samples, 138 (75 %) were tested as positive for HPV; 57 (41.3 %) single HPV infections and 81 (58.7 %) multiple infections. Only 45.6 % of HPV-positive samples presented low-risk genotypes 6 or 11, whereas 71/138 (51.4 %) had at least one oncogenic (high-risk) genotype. Oncogenic genotypes and multiple HPV infections were both associated with a higher number of lifetime sexual partners and their incidence appeared to increase with patient age.

**Conclusions:**

Although it is accepted that HPV 6 and 11 genotypes are main causes of condylomas, our findings show a high incidence of multiple infections and high-risk genotypes in men with benign HPV manifestations. The fact that the condyloma is a skin lesion facilitates the entry of virus into cells and thus cancer progression; therefore, monitoring for HPV is important, especially in those patients with high-risk genotypes (regardless of whether they cause condyloma).

## Background

At present, human papillomavirus (HPV) infections are the most commonly diagnosed sexually transmitted disease [1]. Most infections are asymptomatic and disappear without treatment within a few months [2]. However, some HPV infections can persist for many years causing cell abnormalities, which, if untreated, have the potential to develop into cancer [3].

As infections are sexually transmitted, both men and women can be carriers of HPV. Infections in men usually occur without symptoms, but, unlike women, there are no recommended or approved screening programs for asymptomatic infections in men [4–6]. Furthermore, men often only go to the hospital clinic in the event of a specific concern, being in most cases, a genital wart (condyloma acuminatum).

Ninety percent of condyloma acuminata are caused by two low oncogenic risk genotypes, HPV 6 and 11 [1, 7, 8]. However, as multiple HPV infections with more than one HPV genotype are common and patients are usually sexually active, additional genotypes might also be present on genital warts, including genotypes that are associated with a higher oncogenic risk, such as HPV 16. This genotype has been reported as the third most common genotype detected in condylomas after HPV 6 and HPV 11 in genital warts [7, 9].

In the literature, data on HPV infection prevalence in men vary significantly (1.3 % to 72.9 %) and the exact distribution of specific HPV genotypes remains unclear [10, 11]. Condyloma skin lesions, although benign, facilitate the entry of HPV into cells, and thus increase the risk of cancer development [12]. Therefore, monitoring for the presence of various HPV genotypes might be important, especially in those patients with known high-risk HPV types.

The aim of this study was to assess HPV genotype distribution among men with benign clinical manifestations of HPV-related infections and to evaluate factors associated with HPV infection.

## Methods

### Ethics statement

This study was performed with the approval of Clinical Research Ethics Committee at Basurto University Hospital (Bilbao, Bizkaia, Spain) and all procedures were in accordance with the guidelines and ethical standards for experimental investigation with human subjects required by the Helsinki Declaration of 1975, as revised in 2000.

All patients gave written and informed consent prior their inclusion in the study and parents provided written informed consent on behalf of children (<18 years old) enrolled in our study.

### Recruitment of participants

All men attending the Consultation of Sexually Transmitted Diseases (CSTD) clinic at Basurto University Hospital from 2007 to 2012 due to a lesion in the genital area that urologists suspected as being potentially caused by HPV were eligible for recruitment to the study. No selected groups were used.

### Sample collection

Lesions were tape-stripped to remove possible environmental contaminations [13] and samples were collected by urologists with a saline-wetted Dacron swab device [14]. This device was chosen for sampling due to its greater efficiency compared with cytobrush devices [14]. Sample adequacy was evaluated by amplification of the ß-globin gene with real-time polymerase chain reaction (PCR) [14] and only β-globin positive specimens were included in the study. Samples were stored in their own standard transport medium at -80 °C until they were analysed for HPV presence. The validity of this method for the detection of HPV has been shown previously [14, 15].

Data on medical diagnosis, age at diagnosis, location of lesion, sexual behaviour (type of sexuality) and number of lifetime sexual partners were part of the medical history collected from each individual during the medical consultation at the time of sample collection.

### HPV DNA detection and genotyping

DNA was isolated and molecular genotyping was carried out using a Linear Array HPV Genotyping Test kit (Roche Molecular Diagnostics, Germany), according to manufacturer’s instructions. HPV genotyping was performed by amplifying a fragment of the *L1* gene.

This genotyping kit is capable of identifying up to 37 genotypes. HPV genotypes 16, 18, 31, 33, 35, 39, 45, 51, 52, 56, 58, 59, 68, 73 and 82 were considered to be high-risk types, whereas HPV 26, 53 and HPV 66 were defined as probable high-risk types [16]. All other HPV genotypes were classified as low-risk types.

Men were considered to have a high-risk HPV infection if they were positive for one or more high-risk HPV genotypes – whether or not they were also positive for one or more low-risk HPV genotypes. On the other hand, men were considered to have a low-risk HPV infection if they were positive only to low-risk HPV genotypes.

Genotyping was repeated twice (three times in total) to confirm the result when a multiple genotype HPV infection was detected or a negative result was obtained. Negative and positive controls were provided with the kit and used in every test.

### Statistical analysis

The association between the different variables was determined using the chi-square test. Fisher’s exact test was also performed when the expected numbers were small as it is more accurate than the chi-square test in this situation. SPSS 11.0.1 software (SPSS, Inc. Chicago, IL) was used for the data analysis and a *p*-value below 0.05 was considered to be statistically significant.

Principal Component Analysis (PCA) was performed to compare the genotypes of the HPV infections based on the age, number of lifetime sexual partners and sexual behaviour of the men, In order to do this, men were classified into three age groups (below 30 years, 31–40 years and over 40 years old) and stratified into one of five number of sexual partners groups (<2, 2–5, 6–10 or >10 lifetime sexual partners). Regarding sexual behaviour, a man was categorised as MSM (Men that had Sex with Men) if the participant reported having had sex (anal or oral sex) with another man in his lifetime. All of these PCA analyses were performed using the Unscrambler® software (CAMO).

## Results

A total of 184 β-globin positive anogenital samples were obtained for the study (1 sample per patient) and all specimens were classified by urologists as cauliflower-like growths. No flat lesions or stem-like protrusions were detected.

One hundred and thirty eight specimens (75.0 %) tested positive for HPV (Table 1), including 57 (41.3 %) specimens with a single HPV infection and 81 (58.7 %) specimens with multiple HPV genotype infections. The remaining 46 samples were HPV negative but positive for β-globin.

**Table 1:** Human papillomavirus (HPV) genotypes detected in 138 HPV-positive genital samples

Location	Samples	MSM	Heterosexual	SI	MI	HPV 6,11	LR	HR	OLR	OHR
Penile area										
Foreskin	12	3	9	6	6	5	11	6	6	1
Glans	12	3	9	5	7	0	7	8	4	3
Meatus	12	3	9	3	9	2	6	8	2	6
Shaft	53	22	31	27	26	28	45	25	26	5
Anal area										
Perineum	19	9	10	5	14	11	15	11	8	3
Anus	29	13	16	11	18	16	28	13	15	1
Scrotum	1	1	0	0	1	1	1	0	1	0
**Total**	**138**	**54**	**84**	**57**	**81**	**63**	**113**	**71**	**62**	**19**
**Statistics** ^**a**^										
Chi--square		1.591	1.591	2.081	2.081	3.605	3.038	0.099	0.344	1895
*p*-value		0.21	0.21	0.15	0.15	**0.06**	**0.08**	0.75	0.56	0.17

*HPV 6,11* infections where HPV6 and/or HPV11 were detected, *HR* high-risk genotypes, *LR* low-risk genotypes, *MI* multiple HPV infections, *MSM* men who have sex with other men, *OHR* only high-risk genotypes, *OLR* only low-risk genotypes, *SI* single infection

^a^Statistical association between variables and location of warts considered both penile and anal area total samples and did not take scrotum location into consideration due to small sample size

Low-risk genotypes HPV 6 and/or HPV 11 were detected in 63/138 (45.6 %) of the positive samples. Sixty two of these (44.9 %) showed the presence of only low-risk HPV genotypes and single infections were detected in 45 of these cases (68.1 %). The main HPV genotypes detected in these low-risk infection specimens were: HPV 6 (32/62), 11 (11/62) and 62 (7/62). In contrast, 71 samples (51.4 % of HPV-positive samples) showed the presence of at least one oncogenic (high-risk) HPV genotype, and multiple HPV infection was detected in 60 (84.5 %) of these cases. High-risk HPV predominant types in these samples were: HPV 16 (30/71), 52 (22/71), and 33 (8/71). In this group, the predominant low-risk genotypes associated with high-risk multiple infections were HPVs 6 and 84. The combination of HPV 6 or 11 plus at least one high-risk genotype was detected in 24 specimens.

**Fig. 1:**
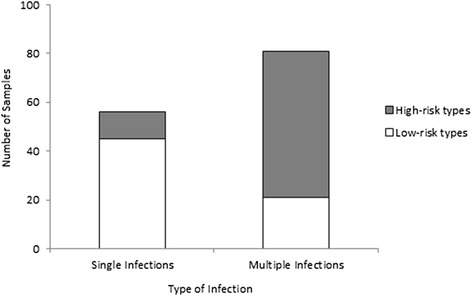
Number of high- and low-risk human papillomavirus (HPV) genotypes by infection type

Comparing the HPV genotypes detected in each type of infection (single *vs*. multiple infection), a significant difference was found (*p* < 0.0001). High-risk genotypes were more frequently found in multiple infections, while low-risk types were more commonly detected in single infections (Fig. 1). However, 19 samples were positive for only high-risk types and 11 of these (57.9 %) were single infections.

### Location of the lesions

The location of the genital warts are shown in Table 1. The presence of single infection, multiple infection, HPV 6 and/or HPV 11, low-risk infection, high-risk infection, and sexual behaviour were all studied for a possible association with wart location. However, scrotum location was not added to the analysis due to small sample size (n = 1).

Overall, multiple infections showed predominance in both the penile area (48 multiple infections *vs*. 41 single infections) and the anal area (32 multiple infections *vs*. 16 single infections) and the proportion of multiple infections seemed to be higher in anal location. However, these results were not statistically significant and could not be used to establish an association between the type of infection and condyloma location.

Men in the MSM group had a higher proportion of anal condylomas than heterosexuals but again statistical analysis did not reveal a significant association. The rest of variables that were analysed did not show any correlation with the location of genital (*p* > 0.1; Table 1).

### Age of the patient

The age of patients ranged from 17 to 72 years. The prevalence of multiple HPV infections tended to increase with age (49 % [25/51] in men aged <30 years, 63.6 % [28/44] in men aged 31–40 years, and 65.1 % [28/43] in men aged >40 years). Similarly, the proportion of patients with at least one high-risk genotype also appeared to increase with age (45 %, 50 %, and 60.5 %, for each age group, respectively); however, none of these differences were considered statistically significant (*p* = 0.208 and *p* = 0.323, respectively; Table 2).

**Table 2:** Distribution of human papillomavirus (HPV) genotypes by age, infection type and oncogenic risk

Age group	Number of people (%)	Single infection				Multiple infection					
		LR	HR	HPV 6,11	Total	LR	HR	HPV 6,11	OLR	OHR	Total
<30	51 (37)	21	5	13	26	19	18	11	7	3	25
30–40	44 (31.9)	13	3	8	16	24	19	14	6	3	28
>40	43 (31.1)	11	3	6	15	25	23	11	4	2	28
**Total**	**138**	**45**	**11**	**27**	**57**	**68**	**60**	**36**	**17**	**8**	**81**
Statistics	*p*-value	0.82	1.00	0.80	0.21	0.40	0.46	0.72	0.472	0.83	0.21
	Chi-square	0.387	0.008	0.443	3.144	1.829	1.569	0.654	1503	0.384	3144

*HPV 6,11* infections where HPV6 and/or HPV11 were detected, *HR* high-risk genotypes, *LR* low-risk genotypes, *OHR* only high-risk genotypes, *OLR* only low-risk genotypes

There was also no statistically significant difference between the age groups with regard to the type of infection (single *vs*. multiple) or risk (high-risk genotypes, HPV 6 and/or HPV 11, low-risk genotypes) at the time of diagnosis (*p* > 0.05; Table 2).

PCA did not reveal any significant difference among the distribution of the different HPV genotypes and age. HPV 40, 67, 69, 70, 26, 68, and 82 were not present in men <30 years, HPV 67 was only detected in men >40 and high-risk genotypes 18 and 31 were found in a total of 5 patients aged >40 while they were not detected in men 31–40 years and only found in one patient aged <30 years.

### Number of sexual partners

**Fig. 2:**
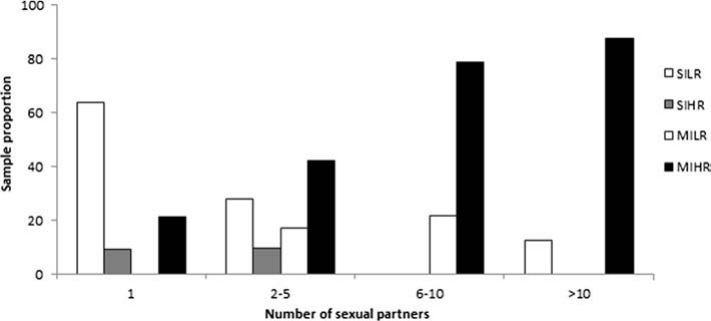
Distribution of oncogenic risk and HPV infection type by number of sexual partners. MILR: multiple HPV infection that presented only low-risk genotypes; MIHR: multiple HPV infection that presented at least one high-risk genotype; SILR: single HPV infection that presented low-risk genotypes; SIHR: single HPV infection that presented high-risk genotypes

The prevalence of multiple HPV infections and high-risk genotypes increased proportionately with the number of lifetime sexual partners with whom the patient had sex (Fig. 2). Single infections and low-risk HPV genotypes were more frequently found in monogamous men while multiple HPV infections and high-risk genotypes were more common in individuals with more than one sexual partner (*p* < 0.01). For example, only 10/33 (30.3 %) with <2 lifetime sexual partners had a high-risk HPV genotype (similar in MSM [29 %] and heterosexuals [31 %]), while this was 43/83 (52 %) in those with 2–5 partners (67 % in MSM and 45 % in heterosexuals; Table 3).

**Table 3:** Prevalence of at least one high-risk human papillomavirus (HPV) genotype by sexual behaviour

	Number of lifetime sexual partners				HPV specific genotypes		
	<2	2–5	6–10	>10	HPV 6	HPV 16	HPV 84
Sexual behaviour							
MSM	2/7 (29)	18/27 (67)	10/12 (83)	7/8 (88 %)	21/54 (39)	18/54 (33)	10/54 (19)
Heterosexual	8/26 (31)	25/56 (45)	1/2 (50)	0	30/84 (36)	12/84 (14)	8/84 (10)
Statistics					Chi square = 0.14	Chi square = 7.01	Chi square = 2.345
					*p* = 0.706	*p* = 0.008	*p* = 0.126

Column for number of lifetime sexual partners shows all high-risk genotypes found in different groups whereas HPV specific genotypes column includes only oncogenic HPV 16 and low-risk HPV 6 and HPV 84 presence in total specimens

*MSM* men that had sex with men

Analysing HPV genotypes individually, PCA revealed that genotypes 6, 16, and 84 were present in a higher proportion among those who had a higher number of lifetime sexual partners. These 3 genotypes were found in 51.5 %, 57.8 %, 64.3 %, and 75 % samples from men that had had <2, 2–5, 6–10, and >10 lifetime sexual partners, respectively. Statistical analysis did not reveal any statistical difference between HPV 6 and 84 prevalence and number of lifetime sexual partners (*p* > 0.05) but for HPV 16 differences were statistically significant. HPV 16 presence was more prevalent as the number of lifetime sexual partners increased (*p* < 0.05; Table 3).

### Sexual behaviour

Fifty four men (39.1 %) confirmed that they had sexual relationships with other men while 84 (60.9 %) confirmed themselves as heterosexual. In general, men in the MSM group had a higher number of sexual partners than heterosexuals and as such the risk of becoming infected with HPV 16 or another high-risk HPV genotype as well as having a multiple infection was higher (Table 3). Multiple infections with more than five HPV genotypes were more frequently found in the MSM group than in heterosexual men (11 samples *vs*. 2 samples).

**Fig. 3:**
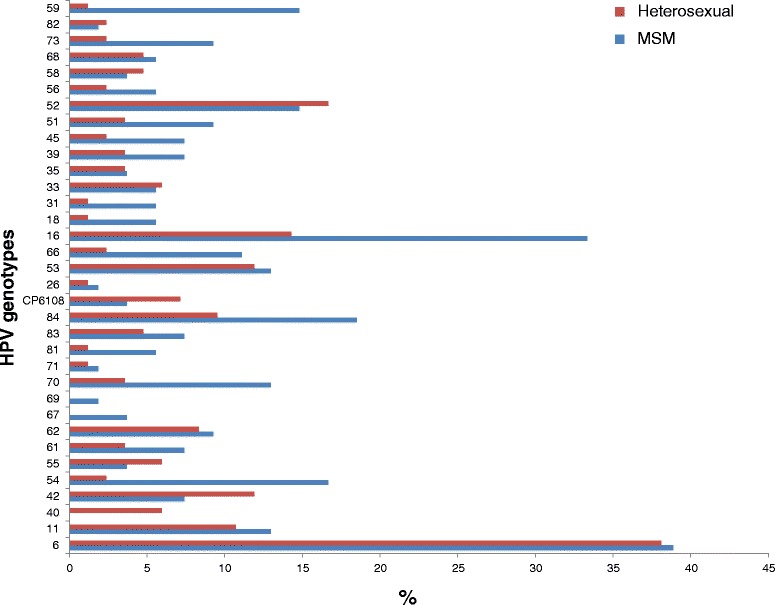
HPV risk genotypes presence and sexual behaviour. Genotypes prevalence proportion. *MSM* Men who have sex with other men

Eight HPV genotypes (HPV 33, 40, 42, 52, 55, 58, 82, and CP6108) were more frequent in heterosexuals than men in the MSM group (Fig. 3), with HPV 40 only detected in heterosexuals. The remaining HPV genotypes were more prevalent in men in the MSM group, with HPV 67 and 69 only present in this group.

PCA analysis revealed a high frequency of HPV 16 and HPV 84 in MSM compared with heterosexuals but only the presence of HPV 16 was significantly different between these groups (*p* < 0.01). Men in the MSM group tended to have a higher risk of being infected with HPV 16 (Table 3).

## Discussion

HPV genotype distribution studies in men are limited, especially in Spain, a country where HPV infection prevalence is reported to be low [17, 18]. There are few reports about HPV prevalence and most of them refer to women. This is the first study carried out in the Basque Country and it indicates that 75 % of men who attended our clinic with genital area clinical manifestations potentially caused by HPV infection, were confirmed to have an HPV infection.

Benign lesions are often caused by low-risk HPV genotypes with genotypes 6 and 11 causing 90 % of genital warts [1, 8]. However, in the present study, low-risk genotypes HPV 6 or HPV 11 were only found in 63/138 (45.6 %) of the samples and 24 of these samples were also positive for at least one high-risk HPV genotype. In this study, the prevalence of multiple HPV infections and high-risk HPV genotypes were high. Multiple HPV infections were more frequent than single infections (58.7 % *vs*. 41.3 %) and 51.4 % of total samples were infected with at least one high-risk HPV genotype. Furthermore, high-risk genotypes were more often detected in multiple infections, while single infections showed a predominance of low-risk genotypes (*p* < 0.01).

Data on the prevalence of HPV infections in men with genital warts is unclear; however, a high prevalence of some high-risk HPV genotypes, such as HPV 16, in asymptomatic men has been reported [19, 20]. In this study, the HPV 16 genotype was the most commonly oncogenic type detected.

It is important to note that these data do not imply that most lesions are caused by high-risk genotypes. It is very likely that low-risk genotypes (HPV 6 and/or 11) are the genotypes responsible for causing the lesion and that high-risk genotypes are present due to patients’ sexual activity. In this study, we showed that presence of HPV 16 was significantly higher in men who had had more lifetime sexual partners.

In our study, HPV DNA was not detected in 46 samples. Negativity with the Linear Array HPV Genotyping Test kit used may be due to specimen inadequacy, genotyping method insensitivity, or the inability of the kit to detect the existence of HPV genotypes other than the 37 genotypes that the kit is capable of identifying. Johansson *et al*. used metagenomic sequencing methods for testing apparently negative HPV condyloma acuminata and found out that most of them were HPV positive [21]. This may also explain why 19 samples in this study were positive for only high-risk HPV genotypes. Sequencing methods would be required in order to confirm whether low-risk genotypes were negative in these samples. Even though HPV is considered to be a significant cause of cancer, there are other important risk factors associated, such as sexual behaviour and number of lifetime sexual partners [22]. We attempted to evaluate these risk factors; however, more data are necessary (especially regarding changes in HPV over time) in order to establish an association between HPV genotype and both viral persistence and progression of HPV-related clinical manifestations.

It is confirmed that prevalence of HPV infection increases with the number of sexual partners [11, 23–27]. Giuliano and Kjaer *et al*. found a strong association between the acquisition of a new HPV infection and the number of sexual partners [23, 26]. In this study, in accordance with the literature, the presence of multiple infections, HPV 16 and overall high-risk HPV genotypes were significantly higher in men with more than one lifetime sexual partner (*p* < 0.05). Men in the MSM group had had more lifetime sexual partners and as such the risk of having HPV 16 or at least one oncogenic HPV genotype as well as a multiple HPV infection were also higher in the MSM group.

According to the results of this study, there was no association between the prevalence of any HPV genotype and age, confirming previous findings [24, 25, 27, 28]; however a trend towards a higher proportion of high-risk genotypes with increased age was seen; a larger data set/sample size may help to confirm or discard the significance of this trend between genotype and age.

These results confirm that HPV infection and reinfection are strongly associated with sexual activity, and support earlier data suggesting that this, and not differences in natural immunity, is the most important factor regarding infection/reinfection risk [29].

There is a wide variation in clearance rates of the various oncogenic HPV genotypes; however, it is well known that the clearance rates for high-risk HPV types are lower than for non-oncogenic types, but clearance of all genotypes increases with age [25] – which may be a factor of decreased sexual activity with age. The trend towards a greater prevalence of oncogenic HPV in older men may be due to the higher persistence of oncogenic HPV genotypes compared with low-risk genotypes.

There are no available approved routine screening tests for genital warts, anogenital cancer, or HPV status in general [4, 6, 10]. In order to accurately estimate HPV genotype prevalence and distribution in men, the detection of the virus in its three manifestations (clinical, subclinical, and latent forms) is required. Estimation of subclinical and latent forms has been carried out by numerous studies assessing HPV prevalence, incidence, and clearance among asymptomatic men and reported HPV prevalence rates vary widely [10, 20, 25]. This may be due to differences in detection and sampling methods, the study population, and geographical locations [5, 10, 30].

One method to estimate HPV prevalence involves collecting specimens from patients who attend hospital clinics with suspected HPV-related conditions, as we have done in this study. However, as infections usually occur without symptoms and most individuals only go to the hospital when a clinical manifestation, such as a genital wart or cancer, is apparent, this method is likely to be inaccurate to estimate HPV general prevalence in men.

More knowledge on the HPV distribution in men is essential for three main reasons: i) the information collected is valuable from the point of view of HPV association with anogenital cancers and genital warts (with follow up studies), ii) the fact that men act as transmitters of HPV infection to both women and men and, iii) to establish a database about the diversity and pathogenicity of different HPV genotypes, which may help to design and optimize treatment and vaccination protocols in order to reduce the disease in men, and consequently in women.

## Conclusion

Although it is accepted that HPV 6 and 11 genotypes are main causes of benign condylomas, our findings show a high incidence of multiple infections and high oncogenic risk genotypes in men with benign HPV manifestations.

## Abbreviations

CSTD: Consultation of Sexually Transmitted Diseases
HPV: human papillomavirus
MSM: men that had sex with men
PCA: principal component analysis
PCR: polymerase chain reaction
